# The Pepper RING Finger E3 Ligase, CaDIR1, Regulates the Drought Stress Response via ABA-Mediated Signaling

**DOI:** 10.3389/fpls.2017.00690

**Published:** 2017-04-28

**Authors:** Hyunhee Joo, Chae Woo Lim, Sang-Wook Han, Sung Chul Lee

**Affiliations:** ^1^Department of Life Science (BK21 Program), Chung-Ang UniversitySeoul, South Korea; ^2^Department of Integrative Plant Science, Chung-Ang UniversityAnseong, South Korea

**Keywords:** abscisic acid, drought, post-translational modification, transpiration, ubiquitination

## Abstract

Drought stress from soil or air limits plant growth and development, leading to a reduction in crop productivity. Several E3 ligases positively or negatively regulate the drought stress response. In the present study, we show that the pepper (*Capsicum annuum*) Drought Induced RING type E3 ligase 1, CaDIR1, regulates the drought stress response via abscisic acid (ABA)-mediated signaling. CaDIR1 contains a C3HC4-type RING finger domain in the N-terminal region; this domain functions during protein degradation via attachment of ubiquitins to the substrate target proteins. The expression levels of the *CaDIR1* gene were suppressed and induced by ABA and drought treatments, respectively. We conducted loss-of-function and gain-of function genetic studies to examine the *in vivo* function of *CaDIR1* in response to ABA and drought stress. *CaDIR1*-silenced pepper plants displayed a drought-tolerant phenotype characterized by a low level of transpirational water loss via increased stomatal closure and elevated leaf temperatures. *CaDIR1-*overexpressing (OX) Arabidopsis plants exhibited an ABA-hypersensitive phenotype during the germination stage, but an ABA-hyposensitive phenotype—characterized by decreased stomatal closure and reduced leaf temperatures—at the adult stage. Moreover, adult *CaDIR1-*OX plants exhibited a drought-sensitive phenotype characterized by high levels of transpirational water loss. Our results indicate that CaDIR1 functions as a negative regulator of the drought stress response via ABA-mediated signaling. Our findings provide a valuable insight into the plant defense mechanism that operates during drought stress.

## Introduction

Plants are sessile organisms; hence, they encounter various environmental stress conditions—including biotic and abiotic stresses. These stresses lead to inhibition of plant growth and development. Water-deficit conditions constitute a major environmental stress and present a serious threat to plant survival. To overcome water-deficit conditions, plants have evolved elaborate adaptive strategies, such as minimizing transpiration water loss from the leaf tissues and maximizing water uptake from the root tissues (Apse and Blumwald, 2002; Yamaguchi-Shinozaki and Shinozaki, 2006; Golldack et al., 2014). Regulation of the transpiration rate via stomatal closure is one of the most effective plant adaptive mechanisms for retaining water. Under drought stress conditions, plant perceive a signal through sensors; this process triggers the expression of defense-related genes and the biosynthesis of the plant hormone abscisic acid (ABA) (Lee and Luan, 2012; Golldack et al., 2014; Lim C. W. et al., 2014). ABA functions in many cellular and physiological processes of plant growth and development, including retardation of seed germination and cotyledon greening. Moreover, ABA plays a crucial role in adaptation to biotic and abiotic stresses—including drought stress—via regulation of various defense-related genes involved in plant survival through modifications of root hydraulic conductivity, osmotic adjustment, and changes in stomatal aperture (Sirichandra et al., 2009; Lim et al., 2015a). Recently, several studies have identified key factors involved in ABA signal transduction from perception to response (Vlad et al., 2009; Ryu et al., 2010; Joseph et al., 2014; Ding et al., 2015). However, the plant defense response via ABA signaling is a complex phenomenon; therefore, the precise functional modifications induced by abiotic stress remain unclear.

Ubiquitination is a unique post-translational modification process in eukaryotes, and it is composed of multiple processes involving the sequential action of three enzymes (Moon et al., 2004; Dreher and Callis, 2007; Stone, 2014). Initially, ubiquitin is activated by E1 (ubiquitin-activating enzyme); next, the activated ubiquitin is transferred to E2 (ubiquitin-conjugating enzyme); and finally, E3 (ubiquitin ligase) recruits and attaches ubiquitin to the substrate target protein (Ciechanover and Schwartz, 1998; Vierstra, 2009; Stone, 2014; Park et al., 2015). Ubiquitination is an intrinsic process involving thousands of distinct E3 ubiquitin ligases, which are critical factors in determining substrate specificity for various target proteins. E3 ubiquitin ligases are classified into two groups based on their subunit compositions. The single subunit subfamily is composed of plant U-box (PUB), homology to E6-AP C-terminus (HECT), and Really Interesting New Gene (RING) types of E3 ligases. On the other hand, the CULLIN4-damaged-specific DNA binding protein1 (CUL4-DDB1) and Skp (S-phase kinase-associated protein)/cullin/F-box (SCF) ligases consist of a multi-subunit (Stone et al., 2005; Pazhouhandeh et al., 2011; Irigoyen et al., 2014; Seo et al., 2014). To date, more than 1,400 E3 ubiquitin ligases have been identified in Arabidopsis (Vierstra, 2009). The Arabidopsis genome encodes more than 470 RING finger domain-containing E3 ubiquitin ligases (Stone et al., 2005; Vierstra, 2009). A number of studies have reported that protein degradation via RING type E3 ubiquitin ligases plays a key role in ABA signaling and abiotic stress responses (Li et al., 2011; Chen et al., 2013). For example, RSL1 (Ring finger of seed longevity 1) is involved in ubiquitination and degradation of PYR1 and PYL4 ABA receptors at the plasma membrane (Bueso et al., 2014). Moreover, RGLGs (Ring domain ligases) are involved in ABA signaling and drought stress responses via regulation of the stability of ABA-signaling components (Cheng et al., 2012; Wu et al., 2016). The functions of E3 ligases in response to abiotic stress via the ABA-signaling pathway have been extensively studied in various plants; however, their precise function remains unclear.

In the present study, we identified and analyzed the RING type E3 ubiquitin ligase, *CaDIR1* (*Capsicum annuum* Drought Induced RING type E3 ligase 1), which contains a RING finger motif. CaDIR1 localized in the nucleus and exhibited *in vivo* E3 ligase activity. We conducted loss-of-function and gain-of-function genetic studies in pepper and Arabidopsis, respectively, to elucidate the *in vivo* functions of CaDIR1. *CaDIR1*-silenced pepper plants displayed a drought-tolerant phenotype characterized by a low level of transpirational water loss. On the other hand, *CaDIR1*-overexpressing (OX) transgenic Arabidopsis plants exhibited a drought-sensitive phenotype. Our data indicate that CaDIR1 functions as a negative regulator of the drought stress response.

## Materials and Methods

### Plant Material and Growth Conditions

Seeds of hot pepper (*C annuum* L. ‘Nockwang’) and tobacco (*Nicotiana benthamiana*) were sown in a steam-sterilized compost soil mix (peat moss, perlite, and vermiculite, 5:3:2, v/v/v) at 27 ± 1°C. The plants were raised under white fluorescent light (80 μmol photons⋅m^-2^⋅s^-1^) with a 16-h light/8-h dark cycle. *Arabidopsis thaliana* ecotype Col-0 seeds were germinated on Murashige and Skoog (1962) (MS) salt supplemented with 0.5% sucrose (Duchefa Biochemie); the plates were incubated in a growth chamber at 24°C. For the phenotypic analysis of response to drought stress, Arabidopsis plants were maintained in a steam-sterilized compost soil mix in a growth chamber under controlled environmental conditions as follows: 24°C and 60% relative humidity under fluorescent light (130 μmol photons⋅m^-2^⋅s^-1^) with a 16-h light/8-h dark cycle.

### Sequence Analysis of the CaDIR1 Protein

The deduced sequences for CaDIR1 and its homologous RING-type ubiquitin E3 ligases were identified using BLAST searches^1^. The SMART^2^ web server was used to identify the RING finger. The amino acid alignment was conducted using ClustalW2^3^, and the results were edited with Genedoc software^4^.

### Virus-Induced Gene Silencing of *CaDIR1*

The virus-induced gene silencing (VIGS) system with the tobacco rattle virus was used to generate *CaDIR1* knockdown pepper plants (Park et al., 2015). The N-terminal region of the *CaDIR1* cDNA (201–434 bp) was inserted into the pTRV2 vector. *Agrobacterium tumefaciens* strain GV3101 containing pTRV1, pTRV2:00, and pTRV:*CaDIR1* was co-infiltrated into the cotyledons of pepper plants (OD_600_ = 0.4 for each construct).

### Generation of *CaDIR1*-OX Transgenic Arabidopsis Plants

The full-length *CaDIR1* cDNA was integrated into the pENTR/D-TOPO vector (Invitrogen, Carlsbad, CA, USA) and was then cloned into the pK2GW7 binary vector using the LR reaction, to induce constitutive expression of the *CaDIR1* gene in Arabidopsis. The 35S:*CaDIR1* construct was introduced into *Agrobacterium tumefaciens* strain GV3101. *Agrobacterium*-mediated transformation of *Arabidopsis thaliana* ecotype Col-0 with the *CaDIR1* gene was conducted using the floral dip method (Clough and Bent, 1998). For selection of *CaDIR1*-OX plants, seeds were plated on MS medium containing 50 μg⋅mL^-1^ kanamycin.

### Subcellular Localization Analysis

The full-length *CaDIR1* cDNA without stop codon were inserted into the p326GFP binary vector. *Agrobacterium tumefaciens* strain GV3101 carrying the 35S:*CaDIR1-GFP* construct was combined with strain p19 (1:1 ratio; OD_600_ = 0.5) and co-infiltrated into the leaves of 4-week-old *N. benthamiana* seedlings. The green fluorescent protein (GFP) signal was observed under a confocal microscope (510 UV/Vis Meta; Zeiss, Oberkochen, Germany) equipped with LSM Image Browser software.

### ABA and Drought Treatments in Pepper and Arabidopsis Plants

For the germination assays, and the measurement of germination rate, primary root growth, and cotyledon greening, 100 seeds per genotype were stratified at 4°C for 3 days and sown on MS agar plates containing various concentrations of ABA. For the post-germination assay, 5-day-old wild-type and *CaDIR1-*OX seedlings grown in the absence of ABA were transferred into MS medium supplemented with 10 μM ABA. After 7 days, the root lengths of the seedlings were measured. The drought stress treatment was performed as described by Lim and Lee (2014). Ten-day-old wild-type and *CaDIR1*-OX seedlings were randomly planted in pots containing soil mixture and maintained under favorable growth conditions. For the drought stress treatment, watering was withheld for 10 days and plants were then re-watered for 1 day. For assessing the transpirational water loss from rosette leaves, 30 leaves were detached from 3-week-old plants and placed in Petri dishes. The dishes were placed in a growth chamber at 40% relative humidity, and the fresh weight was determined 1–7 h after detachment.

### Measurement of Stomatal Aperture

The measurement of stomatal aperture was performed as described previously (Lee et al., 2013) with some modifications. Leaf peels collected from the leaves of 4-week-old pepper plants and 5-week-old Arabidopsis plants were floated in a stomatal opening solution (SOS; 50 mM KCl, 10 mM MES-KOH, 10 μM CaCl_2_, pH 6.15) with light exposure for 3 h. The buffer was replaced with fresh SOS buffer containing various concentrations of ABA. Leaf peels were incubated for an additional 3 h. In each individual sample, 100 stomata were randomly observed under a Nikon Eclipse 80i microscope. The stomatal images were recorded with Image J 1.46r software^5^.

### Measurement of Leaf Temperature

For the measurement of leaf temperature, 4-week-old pepper plants and 5-week-old Arabidopsis plants having fully expanded leaves were treated with 50 μM ABA. Thermal images were obtained using an infrared camera (FLIR systems; T420), and the leaf temperature was measured with FLIR Tools+ ver 5.2 software.

### RNA Isolation and Reverse Transcription-Polymerase Chain Reaction

Total RNA was isolated from the leaf tissues of pepper and Arabidopsis plants using an RNeasy Mini kit (Qiagen, Valencia, CA, USA). The RNA samples were treated with RNA-free DNase to remove genomic DNA. Total RNA (1 μg) was used to synthesize cDNA using a Transcript First Strand cDNA Synthesis kit (Roche, Indianapolis, IN, USA) according to the manufacturer’s instructions. Semi-quantitative and quantitative reverse transcription-polymerase chain (RT-PCR) analyses were performed using Ex-taq (Takara Bio, Shiga, Japan) and iQ^TM^ SYBR Green Supermix (Bio-Rad, Hercules, CA, USA), respectively, with specific primers (Supplementary Table S1). *CaACT1* and *Actin 8* were used as internal controls in pepper and Arabidopsis, respectively.

### Statistical Analyses

To determine significant differences between treatments, statistical analyses were performed using one-way analysis of variance (ANOVA) or Student’s *t*-test. A *P*-value of <0.05 was considered significant.

## Results

### Identification of CaDIR1 as an E3 Ubiquitin Ligase

**FIGURE 1:**
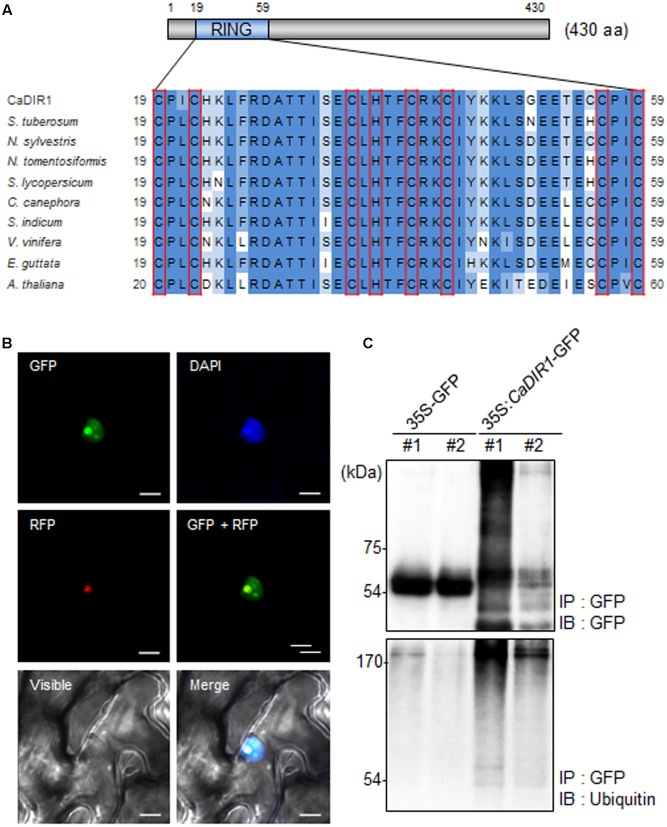
**Amino acid sequence analysis, subcellular localization, and *in vivo* self-ubiquitination of pepper CaDIR1 (*Capsicum annuum* Drought-Induced RING finger protein 1).** **(A)** Alignment of the Really Interesting New Gene (RING) zinc finger C3HC4-type domain. Red boxes indicate conserved cysteine (C) and histidine (H) residues. **(B)** Subcellular localization of CaDIR1 based on transient expression of the green fluorescent protein (GFP) fusion protein in *Nicotiana benthamiana* epidermal cells. The 35S:*CaDIR1-GFP* and 35S:*Fib2-RFP* constructs were expressed using agroinfiltration of *N. benthamiana* leaves and were observed under a confocal laser-scanning microscope. 4′,6-Diamidino-2-phenylindole (DAPI) staining and the Arabidopsis Fib2 protein were used as markers for the nucleus and nucleolus, respectively. White bar = 10 μm. **(C)** *In vivo* self-ubiquitination of CaDIR1. Immunoblot analysis of an *N. benthamiana* leaf harboring and immunoprecipitating the 35S:*CaDIR1-GFP* fusion protein with GFP antibody. Detection of CaDIR1-GFP self-ubiquitination using anti-GFP and anti-ubiquitin antibodies; shifted bands indicate the attachment of ubiquitin molecules.

To isolate novel drought-induced pepper E3 ubiquitin ligase, we performed RNA-seq analysis using pepper leaves that had been subjected to drought stress; we successfully isolated the putative pepper drought-induced candidate E3 ubiquitin ligase (Lim and Lee, 2016). Based on domain analysis and alignment, we designated this gene *CaDIR1* (*C. annuum* Drought Induced RING type E3 ligase 1) (accession no. KY296543). The *CaDIR1* sequence contains a 1293-bp open reading frame, encoding 430 amino acid residues. The mature protein has a molecular mass of 47.9 kDa and an isoelectric point of 9.36. The C3HC4 type RING finger motif, which is essential for E3 ligase in the ubiquitin–26S proteasome system, is located in the N-terminal region of CaDIR1. Multiple sequence alignment analysis revealed that CaDIR1 has relatively high amino acid sequence identity (87.0–87.9%) with other RING type E3 ligases (**Supplementary Figure S1**), especially those containing a RING finger motif (90.3–93.7%) (**Figure 1A**).

Previous studies have reported that several E3 ligases function in the cytoplasm and nucleus (Park et al., 2016; Lim et al., 2017a). To examine the subcellular localization of the CaDIR1 protein in intact cells, the fusion protein of CaDIR1 and the GFP (35S:CaDIR1-GFP) was transiently expressed in *Nicotiana benthamiana* epidermal cells (**Figure 1B**). Expression analysis of the *35S:CaDIR1-GFP* construct revealed that the CaDIR1-GFP fusion protein localized in the nucleus. The blue fluorescent signal for 4′,6-diamidino-2-phenylindole (DAPI) and red fluorescent signal for the fibrillarin-RFP fusion protein were detected in the nucleus and nucleolus, respectively. These results indicate that CaDIR1 functions in the nucleus, especially the nucleolus.

E3 ligases containing RING finger motifs display *in vivo* self-ubiquitination (Liu et al., 2010; Yang et al., 2015). CaDIR1 contains a RING finger motif (**Figure 1A**); hence, we performed an *in vivo* ubiquitination assay to examine whether CaDIR1 functions as an E3 ligase (**Figure 1C**). The *35S:GFP* and *35S:CaDIR1-GFP* constructs were transiently expressed in *N. benthamiana* leaves, and total proteins were isolated. The GFP-tagged proteins were purified, and ubiquitinated proteins were subsequently detected using anti-GFP and anti-ubiquitin antibodies. We found that the CaDIR1-GFP fusion protein was involved in the ubiquitination process in CaDIR1-GFP expressing plant cells.

### Expression Patterns of *CaDIR1* in Pepper Leaves in Response to Abiotic Stresses

**FIGURE 2:**
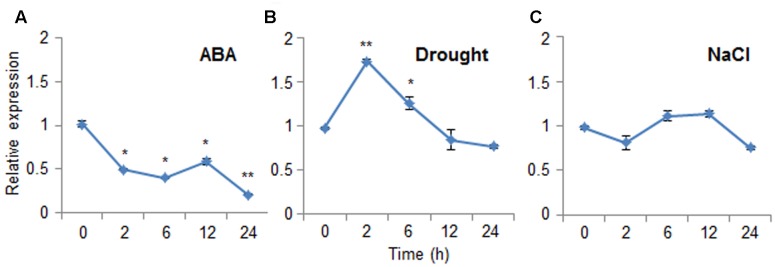
**Expression of the *CaDIR1* gene.** The expression pattern of the *CaDIR1* gene was examined in the leaves of pepper plants after treatment with 100 μM abscisic acid (ABA) **(A)**, drought **(B)**, or 200 mM NaCl **(C)**. The pepper *Actin1* gene was used as an internal control. Data represent the mean ± standard error of three independent experiments. Asterisks indicate significant differences between three independent experiments (Student’s *t*-test; ^∗^*P* < 0.05, ^∗∗^*P* < 0.005).

Abscisic acid functions in the plant response to osmotic stress; moreover, ABA and osmotic stress signals share common components in their signal transduction pathways (Jakab et al., 2005). To investigate the expression patterns of *CaDIR1* in response to ABA and abiotic stresses, we performed Real-Time PCR analysis using leaves harvested from six-leaf stage pepper plants that had been treated with ABA, drought, or NaCl (**Figure 2**). When we monitored the induction of *CaDIR1* transcripts after ABA treatment, the *CaDIR1* transcripts were started to be reduced at 2 h after treatment and continued to be suppressed at 24 h (**Figure 2A**). After drought treatment, the *CaDIR1* transcripts were weakly induced at 2 h and then gradually decreased to the basal level within 12 h (**Figure 2B**). However, high salinity treatment did not significantly altered expression of *CaDIR1* in pepper leaves (**Figure 2C**).

### Enhanced Drought Tolerance of *CaDIR1*-Silenced Pepper Plants

**FIGURE 3:**
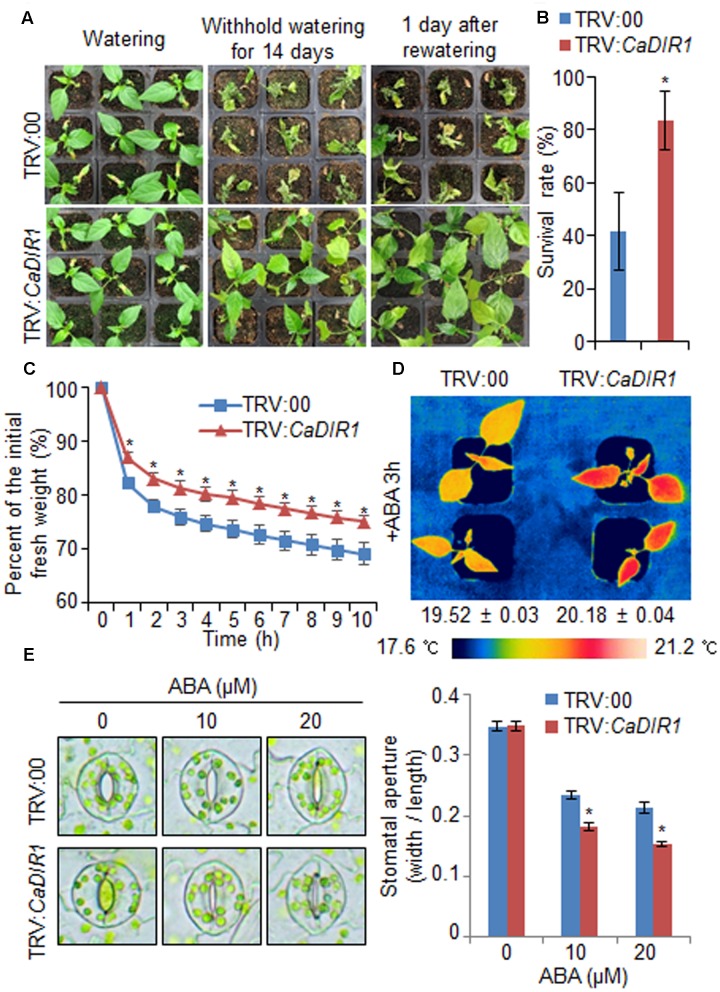
**Enhanced drought tolerance of *CaDIR1-*silenced pepper plants.** **(A)** The drought-tolerant phenotype of *CaDIR1*-silenced pepper plants. Control and *CaDIR1*-silenced pepper plants were grown in pots for 6 weeks under well-watered conditions. Thereafter, watering was withheld for 14 days, followed by re-watering for 1 day. Representative images were taken before (left) and after (middle) drought and after 1 day of re-watering (right). **(B)** Survival rates of control and *CaDIR1*-silenced pepper plants after 1 day of re-watering. Data represent the mean ± standard error of three independent experiments, each evaluating 20 plants. **(C)** Transpirational water loss from the leaves of empty vector control and *CaDIR1*-silenced pepper plants at various times after detachment of leaves. **(D)** Increased leaf temperatures of *CaDIR1-*silenced pepper plants in response to 50 μM ABA treatment. **(E)** Stomatal apertures in control and *CaDIR1*-silenced pepper plants after treatment with various concentrations of ABA. Leaf peels were harvested from 3-week-old plants of each line and incubated in stomatal opening solution (SOS) buffer containing 0, 10, and 20 μM ABA. Representative images were taken under a microscope and the stomatal apertures were measured. Data represent the mean ± standard error of three independent experiments. Asterisks indicate significant differences between three independent experiments (Student’s *t*-test; *P* < 0.05).

To investigate *in vivo* function of CaDIR1, we used VIGS (**Figure 3**). Semi-quantitative RT-PCR analysis revealed that the *CaDIR1* gene was less expressed in *CaDIR1*-silenced pepper plants (TRV:*CaDIR1*) than in control plants (TRV:00) (**Supplementary Figure S2A**); we used these *CaDIR1*-silenced pepper plants in our subsequent phenotypic analyses. First, we examined the function of CaDIR1 in response to drought stress by withholding watering for 14 days and then re-watering for 1 day (**Figure 3A**). Under well-watered conditions, we observed no phenotypic differences between control plants and *CaDIR1-*silenced plants (**Figure 3A**, left panel). However, after withholding watering for 14 days and re-watering for 1 day, control plants showed a more wilted phenotype than *CaDIR1*-silenced plants (**Figure 3A**, middle and right panels). Moreover, after re-watering, the survival rate of *CaDIR1*-silenced plants was 83%, whereas that of control plants was approximately 41% (**Figure 3B**). To evaluate whether the drought-tolerant phenotype displayed by *CaDIR1*-silenced pepper plants was derived from enhanced capacity for water retention, we measured the transpirational water loss of detached pepper leaves (**Figure 3C**). At various time points after detachment, the leaf fresh weight was significantly higher in *CaDIR1*-silenced plants (75%) than in control plants (69%). Previous reports have suggested that altered water retention is associated with ABA sensitivity (Cheong et al., 2007; Santiago et al., 2009; Ryu et al., 2010; Lim et al., 2015c); hence, we monitored the leaf temperatures and stomatal apertures after treatment with ABA (**Figures 3D,E**). The leaf temperatures of *CaDIR1*-silenced pepper plants were higher than those of control plants (**Figure 3D**). Stomatal movement leads to an increase in evaporative cooling, and this influences the leaf temperature. Hence, we assessed the stomatal apertures after treatment with various concentrations of ABA. Consistent with the leaf temperature data, the stomatal apertures of *CaDIR1-*silenced plants were smaller than those of control plants (**Figure 3E**).

### Altered ABA Sensitivity of *CaDIR1*-OX Transgenic Arabidopsis Plants at Different Growth Stages

*CaDIR1*-silenced pepper plants displayed a drought-tolerant phenotype (**Figure 3**). Therefore, we performed additional reverse genetic analyses to evaluate the *in vivo* function of CaDIR1 in response to abiotic stress. We generated 35S:*CaDIR1* Arabidopsis transgenic plants in the Col-0 ecotype background; these plants showed overexpression of the *CaDIR1* gene. Semi-quantitative reverse transcription-polymerase chain reaction (RT-PCR) analysis revealed the expression of *CaDIR1* transcripts in two independent T_3_ homozygous transgenic Arabidopsis lines, but not in wild-type plants (**Supplementary Figure 2B**). We used these *CaDIR1*-overexpressing (OX) plants in our subsequent phenotypic analyses.

**FIGURE 4:**
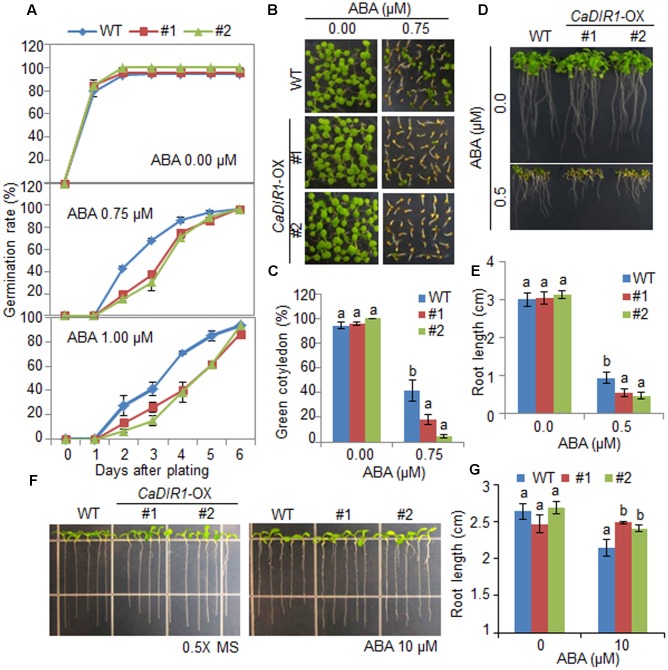
**Enhanced and reduced sensitivity of *CaDIR1-*overexpressing (OX) transgenic Arabidopsis plants to ABA during the germinative and post-germinative growth stages, respectively.** **(A)** Seed germination of wild-type (WT) and *CaDIR1-*OX plants in response to ABA. Seeds were germinated on 0.5x Murashige and Skoog (MS) medium containing 0.0, 0.75, or 1.0 μM ABA. **(B,C)** Seedling development of WT and *CaDIR1*-OX plants exposed to ABA. Representative photographs were taken 5 days after plating **(B)**. Quantification of green cotyledons in WT and transgenic plants was performed 5 days after plating **(C)**. Data represent the mean ± standard error values obtained after evaluating 72 seeds from three independent experiments. **(D,E)** Primary root elongation of WT and transgenic plants in response to ABA. The root length of each plant was measured 8 days after plating. **(F,G)** Primary root elongation of WT and transgenic plants exposed to ABA after germination. Five-day-old seedlings grown on 0.5x MS medium were transferred to fresh 0.5x MS medium containing 0 μM or 10 μM ABA. After 7 days, the representative images were taken **(F)**, and the root length in each line was measured **(G)**. Data represent the mean ± standard error of three independent experiments. Different letters indicate significant differences between three independent experiments (ANOVA; *P* < 0.05).

To elucidate the involvement of CaDIR1 in ABA signaling, we conducted phenotypic analysis of *CaDIR1*-OX plants at the germinative and post-germinative stages in response to ABA (**Figure 4**). First, we germinated *CaDIR1*-OX seeds on Murashige and Skoog (MS) medium supplemented with 0, 0.75, and 1.00 μM ABA. In the absence of ABA, we determined no significant difference in germination rates between wild-type and *CaDIR1*-OX seeds. However, in the presence of ABA, the germination rate of *CaDIR1*-OX seeds was significantly lower than that of wild-type seeds (**Figure 4A**). Next, we examined seedling establishment and root growth of wild-type and *CaDIR1-*OX plants in response to ABA (**Figures 4B–E**). Consistent with the germination rate, the rate of cotyledon greening and primary root growth were significantly lower in *CaDIR1*-OX plants than in wild-type plants. To determine whether the altered ABA sensitivity of *CaDIR1*-OX plants was derived indirectly from the influence of ABA on seed germination or directly from the influence of ABA on seedling growth, 5-day-old seedlings germinated on MS medium were transferred to fresh MS medium supplemented with 0 μM or 10 μM ABA (**Figures 4F,G**). Contrary to our germination data, the roots of *CaDIR1*-OX seedlings were significantly longer than those of wild-type seedlings, indicating that the altered ABA sensitivity displayed by *CaDIR1*-OX plants is dependent on the growth stage.

**FIGURE 5:**
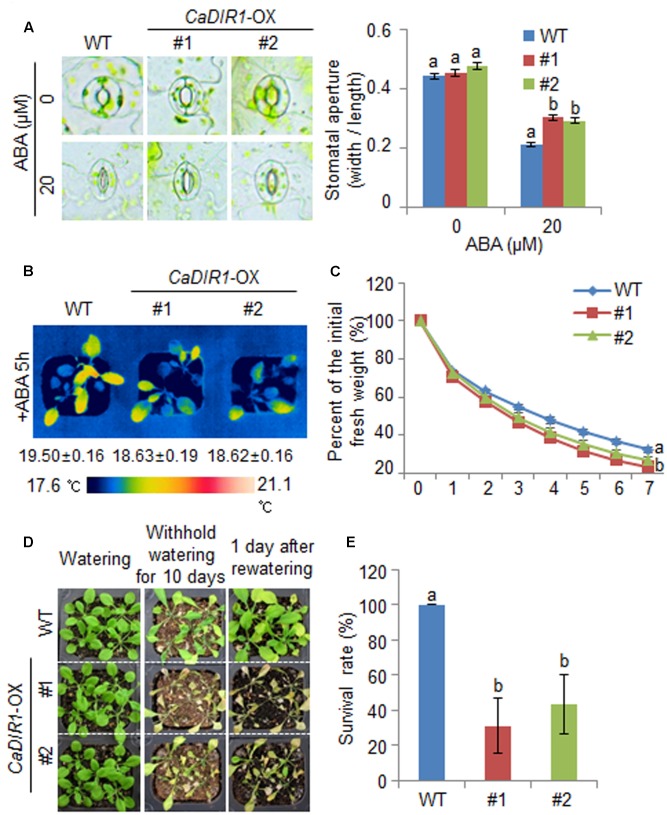
**∣ Reduced tolerance of *CaDIR1-*OX plants to drought stress.** **(A)** Stomatal apertures in wild-type (WT) and *CaDIR1-*OX plants treated with ABA. Leaf peels were harvested from the 3-week-old plants of each line and incubated in SOS buffer containing 0 μM or 20 μM ABA. Representative images were taken under a microscope and the stomatal apertures were measured. Data represent the mean ± standard error of three independent experiments. **(B)** Decreased leaf temperatures of *CaDIR1-*OX plants in response to 50 μM ABA treatment. **(C)** Transpirational water loss from the leaves of WT and transgenic plants at various times after detachment of leaves. **(D)** Drought-sensitive phenotype of *CaDIR1-*OX plants. Four-week-old WT and transgenic plants were subjected to drought stress by withholding watering for 10 days and then re-watering for 1 day. Representative images were taken before (left) and after (middle) drought and after 1 day of re-watering (right). **(E)** Survival rates of plants after 1 day of re-watering. Data represent the mean ± standard error of three independent experiments, each evaluating 20 plants.

We further examined the altered phenotypes of adult wild-type and *CaDIR1*-OX plants in response to ABA by measuring the stomatal apertures and leaf temperatures (**Figures 5A,B**). In the absence of ABA, we determined no significant differences in stomatal apertures or leaf temperatures between wild-type and *CaDIR1*-OX plants. However, after exposure to 20 μM ABA, the stomatal apertures of *CaDIR1*-OX plants were significantly larger than those of wild-type plants (**Figure 5A**). Moreover, after exposure to 50 μM ABA, the leaf temperatures of *CaDIR1*-OX plants were significantly lower than those of wild-type plants (**Figure 5B**). Our results indicate that *CaDIR1*-OX plants exhibit altered responses to ABA in a growth-stage dependent manner.

### Reduced Drought Tolerance of *CaDIR1*-OX Transgenic Plants

To investigate whether the ABA-hyposensitive phenotype displayed by adult *CaDIR1-*OX plants influences altered water retention, we assessed the transpirational water loss by measuring the fresh weight of detached rosette leaves (**Figure 5C**). In the presence of ABA, the fresh weight of *CaDIR1-*OX leaves was significantly lower than that of wild-type leaves. To investigate the influence of *CaDIR1* overexpression on drought tolerance, we conducted phenotypic analysis of wild-type and *CaDIR1*-OX plants in response to drought stress (**Figure 5D**). Under well-watered conditions, we observed no phenotypic differences between wild-type and *CaDIR1*-OX plants (**Figure 5D**, left panel). However, when we subjected plants to drought stress by withholding watering for 10 days and then re-watering for 1 day, *CaDIR1*-OX plants exhibited more wilted phenotypes than wild-type plants (**Figure 5D**, middle and right panels). Moreover, after re-watering, 100% of wild-type plants resumed their growth, whereas only 31–43% of the *CaDIR1*-OX plants survived (**Figure 5E**). Our results indicate that the reduced capacity for water retention of *CaDIR1*-OX plants is derived from ABA hyposensitivity, and this contributes to a drought-sensitive phenotype.

**FIGURE 6:**
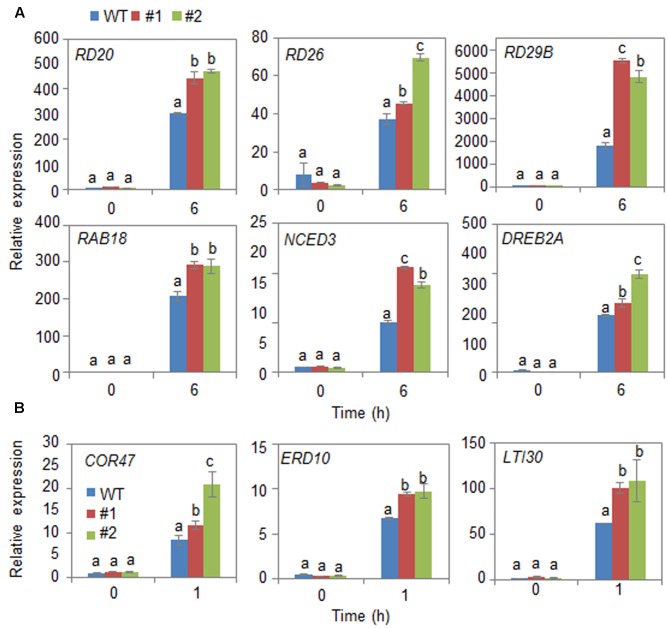
**Quantitative reverse transcription-polymerase chain reaction (qRT-PCR) analysis of drought stress-inducible genes in *CaDIR1*-OX leaves exposed to drought stress through detachment** **(A)** and 50 μM ABA treatment **(B)**. The relative expression (ΔΔCT) level of each gene was normalized to that of *Actin 8* as an internal control gene. Data represent the mean ± standard deviation of three independent experiments. Different letters indicate significant differences between three independent experiments (ANOVA; *P* < 0.05 followed by Fisher’s LSD test).

Next, we examined the mechanism whereby *CaDIR1* overexpression influences ABA biosynthesis and drought stress signaling (**Figure 6**). We performed qPCR analysis of wild-type and *CaDIR1*-OX leaves that had been subjected to drought stress through detachment. We found that after 6 h of drought stress treatment, the expression levels of stress-responsive genes—including *NCED3*, *DREB2A*, *RD29B*, *RD20*, *RD26*, and *RAB18*—were significantly higher in *CaDIR1*-OX leaves than in wild-type leaves (**Figure 6A**). Moreover, the dehydrin genes, including *COR47*, *ERD10*, and *LTI30*, were also more induced in *CaDIR1*-OX leaves than in wild-type leaves at 1 h after ABA treatment (**Figure 6B**). Our data indicate that CaDIR1 negatively regulates drought tolerance in Arabidopsis and pepper plants by modulating ABA-mediated stomatal closure.

## Discussion

In the present study, we isolated a drought stress-inducible RING type E3 ligase gene, *CaDIR1*, which functions as a negative regulator of the drought stress response via ABA-dependent signal transduction. Protein degradation via the ubiquitin–proteasome system plays an important role in regulating the plant response to abiotic stress (Lee et al., 2011; Guo et al., 2013). Several abiotic stress-related E3 ligases have been isolated and functionally characterized; nevertheless, the precise molecular and physiological mechanisms whereby plants adapt to abiotic stress remain unclear. Post-translational degradation via the ubiquitin–proteasome system facilitates rapid adaptation to variable environmental conditions through ABA-mediated signaling (Lyzenga et al., 2012). Under water-deficit conditions—such as drought stress—ABA biosynthesis is increased in various plant tissues and accumulates in the leaf tissues, especially the guard cells (Zhu, 2002; Cutler et al., 2010; Hubbard et al., 2010). Induction of ABA leads to increased expression of defense-related genes, and this induces stomatal closure and contributes to drought tolerance (Robertson and Chandler, 1994; Murata et al., 2015; Park et al., 2015). A number of studies have reported that under drought stress conditions, RING type E3 ligases—such as RGLG1, RGLG5, SDIR1, OsCTR1, XERICO, Rha2a, and Rha2b—function as positive regulators of ABA (Ko et al., 2006; Li et al., 2011; Lim S. D. et al., 2014; Zhang et al., 2015; Wu et al., 2016). In contrast, under normal condition, RING type E3 ligases—including RSL1, RGLG2, and AIP2—negatively regulate ABA signaling, and this affect protein stability of positive regulators of ABA, such as ABA receptors and transcription factors (Cheng et al., 2012; Bueso et al., 2014; Zhang et al., 2015). The results of our present phenotypic analysis and *in vivo* ubiquitination assay imply that CaDIR1 is involved in the degradation of target proteins, which act as positive regulators of the drought stress response.

The expression levels of ABA biosynthesis- and/or defense-related genes are critical to the plant defense response to drought stress (Zhang et al., 2006; Aubert et al., 2010; Hubbard et al., 2010; Fujita et al., 2011; Lim et al., 2015b). Here, we used *CaDIR1*-OX transgenic Arabidopsis plants to elucidate the *in vivo* function of CaDIR1. The *CaDIR1*-OX plants have different ABA phenotypes depending on its developmental stages. These reversed phenotypes suggested that the function of CaDIR1 is different in response to stress on developmental stages. Adult *CaDIR1*-OX transgenic Arabidopsis plants displayed ABA-hyposensitive phenotypes characterized by decreased stomatal closure and reduced leaf temperatures (**Figure 5**). Under drought stress conditions, the expression of *NCED3* is induced and ABA biosynthesis in plant tissues is increased, leading to amplification of ABA-dependent signaling (Iuchi et al., 2001). Several studies have demonstrated that the expression levels of defense-related genes are closely related to abiotic stress tolerance (Verslues and Bray, 2006; Shinozaki and Yamaguchi-Shinozaki, 2007; Aubert et al., 2010). Our *CaDIR1*-OX plants displayed a drought-sensitive phenotype; therefore, we predicted that these plants would show low expression levels of defense-related genes. Contrary to our prediction, the expression levels of defense-related genes—including *NCED3*, *DREB2A*, and *RD29B*—were higher in *CaDIR1*-OX plants than in wild-type plants. We propose that if *CaDIR1*-OX plants lack the facility to induce a successful defense response, they cannot alleviate drought stress signals; hence, these stress signals are continually transferred to the plant tissues—especially the leaf tissue—leading to enhanced expression of defense-related genes, including *NCED3* (Lim et al., 2017b). Moreover, *NCED3* positively regulates the transcription of defense-related genes (Urano et al., 2009); hence, up-regulation of *NCED3* influences the expression of defense-related genes. In the ABA-mediated drought stress response, CaDIR1 regulates the expression levels of defense-related genes directly or indirectly; however, the precise mechanisms underlying this regulatory process remain unclear.

## Conclusion

We have demonstrated that the RING-type E3 ubiquitin ligase CaDIR1 negatively regulates the plant defense response to drought stress in adult pepper plants via ABA-mediated signaling. In our gain-of-function and loss-of-function genetic studies, *CaDIR1-*OX Arabidopsis plants and *CaDIR1*-silenced pepper plants exhibited drought-sensitive and drought-tolerant phenotypes, respectively, and these phenotypes were associated with altered responses to ABA. We were unable to identify the E3 ligase target proteins, which presumably function downstream of CaDIR1. Further studies to identify the downstream target proteins regulated by CaDIR1 E3 ligase are required. Our findings provide a valuable insight into the plant defense response to drought stress via the ABA-mediated signaling pathway.

## Author Contributions

HJ and CWL performed experiments and analyzed the results. S-WH and SCL designed the experiments and wrote the manuscript.

## Conflict of Interest Statement

The authors declare that the research was conducted in the absence of any commercial or financial relationships that could be construed as a potential conflict of interest. The handling Editor declared a past co-authorship with one of the authors SCL and states that the process nevertheless met the standards of a fair and objective review.

## Footnotes

1 http://www.ncbi.nlm.nih.gov/BLAST

2 http://smart.embl-heidelberg.de/

3 http://www.ebi.ac.uk/Tools/msa/clustalw2

4 http://www.nrbsc.org/gfx/genedoc

5 http://imagej.nih.gov/ij

## Supplementary Material

**FIGURE S1 | (A)** Multiple alignment of amino acids in the CaDIR1 protein and its homologous RING type E3 ligases was performed using ClustalW2. **(B)** Phylogenetic tree of CaDIR1 and other plant RING finger type E3 ligases. The tree was constructed using the amino acid sequences indexed in GenBank BLAST database. The length of each pair of branches represents the distance between sequence pairs.

**FIGURE S2 | (A)** RT-PCR analysis of *CaDIR1* gene expression in the leaves of *CaDIR1*-silenced pepper plants (TRV:*CaDIR1*) and empty vector control pepper plants (TRV:00) 24 h after treatment with ABA. *CaACT1* was used as an internal control gene. **(B)** RT-PCR analysis of *CaDIR1* expression in wild-type plants and *CaDIR1-*OX transgenic lines. *Actin8* was used as an internal control gene.

## References

[B1] Apse M. P. Blumwald E. (2002). Engineering salt tolerance in plants. *Curr. Opin. Biotechnol.* 13 146–150. 10.1016/S0958-1669(02)00298-7 11950567

[B2] Aubert Y. Vile D. Pervent M. Aldon D. Ranty B. Simonneau T. (2010). RD20, a stress-inducible caleosin, participates in stomatal control, transpiration and drought tolerance in *Arabidopsis thaliana*. *Plant Cell Physiol.* 51 1975–1987. 10.1093/pcp/pcq155 20952421

[B3] Bueso E. Rodriguez L. Lorenzo-Orts L. Gonzalez-Guzman M. Sayas E. Munoz-Bertomeu J. (2014). The single-subunit RING-type E3 ubiquitin ligase RSL1 targets PYL4 and PYR1 ABA receptors in plasma membrane to modulate abscisic acid signaling. *Plant J.* 80 1057–1071. 10.1111/tpj.12708 25330042

[B4] Chen Y. T. Liu H. Stone S. Callis J. (2013). ABA and the ubiquitin E3 ligase KEEP ON GOING affect proteolysis of the *Arabidopsis thaliana* transcription factors ABF1 and ABF3. *Plant J.* 75 965–976. 10.1111/tpj.12259 23742014 PMC3823012

[B5] Cheng M. C. Hsieh E. J. Chen J. H. Chen H. Y. Lin T. P. (2012). Arabidopsis RGLG2, functioning as a RING E3 ligase, interacts with AtERF53 and negatively regulates the plant drought stress response. *Plant Physiol.* 158 363–375. 10.1104/pp.111.189738 22095047 PMC3252077

[B6] Cheong Y. H. Pandey G. K. Grant J. J. Batistic O. Li L. Kim B. G. (2007). Two calcineurin B-like calcium sensors, interacting with protein kinase CIPK23, regulate leaf transpiration and root potassium uptake in Arabidopsis. *Plant J.* 52 223–239. 10.1111/j.1365-313X.2007.03236.x 17922773

[B7] Ciechanover A. Schwartz A. L. (1998). The ubiquitin-proteasome pathway: the complexity and myriad functions of proteins death. *Proc. Natl. Acad. Sci. U.S.A.* 95 2727–2730. 10.1073/pnas.95.6.2727 9501156 PMC34259

[B8] Clough S. J. Bent A. F. (1998). Floral dip: a simplified method for *Agrobacterium*-mediated transformation of *Arabidopsis thaliana*. *Plant J.* 16 735–743. 10.1046/j.1365-313x.1998.00343.x 10069079

[B9] Cutler S. R. Rodriguez P. L. Finkelstein R. R. Abrams S. R. (2010). Abscisic acid: emergence of a core signaling network. *Annu. Rev. Plant Biol.* 61 651–679. 10.1146/annurev-arplant-042809-112122 20192755

[B10] Ding Y. Li H. Zhang X. Xie Q. Gong Z. Yang S. (2015). OST1 kinase modulates freezing tolerance by enhancing ICE1 stability in *Arabidopsis*. *Dev. Cell* 32 278–289. 10.1016/j.devcel.2014.12.023 25669882

[B11] Dreher K. Callis J. (2007). Ubiquitin, hormones and biotic stress in plants. *Ann. Bot.* 99 787–822. 10.1093/aob/mcl255 17220175 PMC2802907

[B12] Fujita Y. Fujita M. Shinozaki K. Yamaguchi-Shinozaki K. (2011). ABA-mediated transcriptional regulation in response to osmotic stress in plants. *J. Plant Res.* 124 509–525. 10.1007/s10265-011-0412-3 21416314

[B13] Golldack D. Li C. Mohan H. Probst N. (2014). Tolerance to drought and salt stress in plants: unraveling the signaling networks. *Front. Plant Sci.* 5:151 10.3389/fpls.2014.00151 PMC4001066 24795738

[B14] Guo L. Nezames C. D. Sheng L. Deng X. Wei N. (2013). Cullin-RING ubiquitin ligase family in plant abiotic stress pathways(F). *J. Integr. Plant Biol.* 55 21–30. 10.1111/jipb.12019 23206256

[B15] Hubbard K. E. Nishimura N. Hitomi K. Getzoff E. D. Schroeder J. I. (2010). Early abscisic acid signal transduction mechanisms: newly discovered components and newly emerging questions. *Genes Dev.* 24 1695–1708. 10.1101/gad.1953910 20713515 PMC2922499

[B16] Irigoyen M. L. Iniesto E. Rodriguez L. Puga M. I. Yanagawa Y. Pick E. (2014). Targeted degradation of abscisic acid receptors is mediated by the ubiquitin ligase substrate adaptor DDA1 in *Arabidopsis*. *Plant Cell* 26 712–728. 10.1105/tpc.113.122234 24563205 PMC3967035

[B17] Iuchi S. Kobayashi M. Taji T. Naramoto M. Seki M. Kato T. (2001). Regulation of drought tolerance by gene manipulation of 9-cis-epoxycarotenoid dioxygenase, a key enzyme in abscisic acid biosynthesis in Arabidopsis. *Plant J.* 27 325–333. 10.1046/j.1365-313x.2001.01096.x 11532178

[B18] Jakab G. Ton J. Flors V. Zimmerli L. Metraux J. P. Mauch-Mani B. (2005). Enhancing Arabidopsis salt and drought stress tolerance by chemical priming for its abscisic acid responses. *Plant Physiol.* 139 267–274. 10.1104/pp.105.065698 16113213 PMC1203376

[B19] Joseph M. P. Papdi C. Kozma-Bognar L. Nagy I. Lopez-Carbonell M. Rigo G. (2014). The Arabidopsis ZINC FINGER PROTEIN3 interferes with abscisic acid and light signaling in seed germination and plant development. *Plant Physiol.* 165 1203–1220. 10.1104/pp.113.234294 24808098 PMC4081332

[B20] Ko J. H. Yang S. H. Han K. H. (2006). Upregulation of an Arabidopsis RING-H2 gene, *XERICO*, confers drought tolerance through increased abscisic acid biosynthesis. *Plant J.* 47 343–355. 10.1111/j.1365-313X.2006.02782.x 16792696

[B21] Lee D. H. Choi H. W. Hwang B. K. (2011). The pepper E3 ubiquitin ligase RING1 gene, CaRING1, is required for cell death and the salicylic acid-dependent defense response. *Plant Physiol.* 156 2011–2025. 10.1104/pp.111.177568 21628629 PMC3149946

[B22] Lee S. C. Lim C. W. Lan W. He K. Luan S. (2013). ABA signaling in guard cells entails a dynamic protein-protein interaction relay from the PYL-RCAR family receptors to ion channels. *Mol. Plant* 6 528–538. 10.1093/mp/sss078 22935148

[B23] Lee S. C. Luan S. (2012). ABA signal transduction at the crossroad of biotic and abiotic stress responses. *Plant Cell Environ.* 35 53–60. 10.1111/j.1365-3040.2011.02426.x 21923759

[B24] Li H. Jiang H. Bu Q. Zhao Q. Sun J. Xie Q. (2011). The Arabidopsis RING finger E3 ligase RHA2b acts additively with RHA2a in regulating abscisic acid signaling and drought response. *Plant Physiol.* 156 550–563. 10.1104/pp.111.176214 21478367 PMC3177258

[B25] Lim C. W. Baek W. Lee S. C. (2017a). The pepper RING-type E3 ligase CaAIRF1 regulates ABA and drought signaling via CaADIP1 protein phosphatase degradation. *Plant Physiol.* 173 2323–2339. 10.1104/pp.16.01817 28184010 PMC5373060

[B26] Lim C. W. Park C. Kim J. H. Joo H. Hong E. Lee S. C. (2017b). Pepper CaREL1, a ubiquitin E3 ligase, regulates drought tolerance via the ABA-signalling pathway. *Sci. Rep.* 7:477 10.1038/s41598-017-00490-4 PMC5428412 28352121

[B27] Lim C. W. Baek W. Lim S. Han S. W. Lee S. C. (2015a). Expression and functional roles of the pepper pathogen-induced bZIP transcription factor CabZIP2 in enhanced disease resistance to bacterial pathogen infection. *Mol. Plant Microbe Interact.* 28 825–833. 10.1094/MPMI-10-14-0313-R 25738319

[B28] Lim C. W. Han S. W. Hwang I. S. Kim D. S. Hwang B. K. Lee S. C. (2015b). The pepper lipoxygenase CaLOX1 plays a role in osmotic, drought and high salinity stress response. *Plant Cell Physiol.* 56 930–942. 10.1093/pcp/pcv020 25657344

[B29] Lim C. W. Hwang B. K. Lee S. C. (2015c). Functional roles of the pepper RING finger protein gene, CaRING1, in abscisic acid signaling and dehydration tolerance. *Plant Mol. Biol.* 89 143–156. 10.1007/s11103-015-0359-1 26249046

[B30] Lim C. W. Lee S. C. (2014). Functional roles of the pepper MLO protein gene, CaMLO2, in abscisic acid signaling and drought sensitivity. *Plant Mol. Biol.* 85 1–10. 10.1007/s11103-013-0155-8 24282068

[B31] Lim C. W. Lee S. C. (2016). Pepper protein phosphatase type 2C, CaADIP1 and its interacting partner CaRLP1 antagonistically regulate ABA signalling and drought response. *Plant Cell Environ.* 39 1559–1575. 10.1111/pce.12721 26825039

[B32] Lim C. W. Luan S. Lee S. C. (2014). A prominent role for RCAR3-mediated ABA signaling in response to *Pseudomonas syringae* pv. *tomato* DC3000 infection in Arabidopsis. *Plant Cell Physiol.* 55 1691–1703. 10.1093/pcp/pcu100 25063782

[B33] Lim S. D. Lee C. Jang C. S. (2014). The rice RING E3 ligase, OsCTR1, inhibits trafficking to the chloroplasts of OsCP12 and OsRP1, and its overexpression confers drought tolerance in *Arabidopsis*. *Plant Cell Environ.* 37 1097–1113. 10.1111/pce.12219 24215658

[B34] Liu L. Zhang Y. Tang S. Zhao Q. Zhang Z. Zhang H. (2010). An efficient system to detect protein ubiquitination by agroinfiltration in *Nicotiana benthamiana*. *Plant J.* 61 893–903. 10.1111/j.1365-313X.2009.04109.x 20015064

[B35] Lyzenga W. J. Booth J. K. Stone S. L. (2012). The Arabidopsis RING-type E3 ligase XBAT32 mediates the proteasomal degradation of the ethylene biosynthetic enzyme, 1-aminocyclopropane-1-carboxylate synthase 7. *Plant J.* 71 23–34. 10.1111/j.1365-313X.2012.04965.x 22339729

[B36] Moon J. Parry G. Estelle M. (2004). The ubiquitin-proteasome pathway and plant development. *Plant Cell* 16 3181–3195. 10.1105/tpc.104.161220 15579807 PMC535867

[B37] Murashige T. Skoog F. (1962). A revised medium for rapid growth and bio-assays with tobacco tissue cultures. *Physiol. Plant.* 15 473–497. 10.1111/j.1399-3054.1962.tb08052.x

[B38] Murata Y. Mori I. C. Munemasa S. (2015). Diverse stomatal signaling and the signal integration mechanism. *Annu. Rev. Plant Biol.* 66 369–392. 10.1146/annurev-arplant-043014-114707 25665132

[B39] Park C. Lim C. W. Baek W. Lee S. C. (2015). RING type E3 ligase CaAIR1 in pepper acts in the regulation of ABA signaling and drought stress response. *Plant Cell Physiol.* 56 1808–1819. 10.1093/pcp/pcv103 26169196

[B40] Park C. Lim C. W. Lee S. C. (2016). The pepper RING-Type E3 ligase, CaAIP1, functions as a positive regulator of drought and high salinity stress responses. *Plant Cell Physiol.* 57 2202–2212. 10.1093/pcp/pcw139 27503217

[B41] Pazhouhandeh M. Molinier J. Berr A. Genschik P. (2011). MSI4/FVE interacts with CUL4-DDB1 and a PRC2-like complex to control epigenetic regulation of flowering time in *Arabidopsis*. *Proc. Natl. Acad. Sci. U.S.A.* 108 3430–3435. 10.1073/pnas.1018242108 21282611 PMC3044392

[B42] Robertson M. Chandler P. M. (1994). A dehydrin cognate protein from pea (*Pisum sativum* L.) with an atypical pattern of expression. *Plant Mol. Biol.* 26 805–816. 10.1093/pcp/pcv103 7999996

[B43] Ryu M. Y. Cho S. K. Kim W. T. (2010). The Arabidopsis C3H2C3-type RING E3 ubiquitin ligase AtAIRP1 is a positive regulator of an abscisic acid-dependent response to drought stress. *Plant Physiol.* 154 1983–1997. 10.1104/pp.110.164749 20884812 PMC2996028

[B44] Santiago J. Rodrigues A. Saez A. Rubio S. Antoni R. Dupeux F. (2009). Modulation of drought resistance by the abscisic acid receptor PYL5 through inhibition of clade A PP2Cs. *Plant J.* 60 575–588. 10.1111/j.1365-313X.2009.03981.x 19624469

[B45] Seo I. Lee J. H. Nezames C. D. Zhong S. Song E. Byun M. O. (2014). ABD1 is an *Arabidopsis* DCAF substrate receptor for CUL4-DDB1-based E3 ligases that acts as a negative regulator of abscisic acid signaling. *Plant Cell* 26 695–711. 10.1105/tpc.113.119974 24563203 PMC3967034

[B46] Shinozaki K. Yamaguchi-Shinozaki K. (2007). Gene networks involved in drought stress response and tolerance. *J. Exp. Bot.* 58 221–227. 10.1093/jxb/erl164 17075077

[B47] Sirichandra C. Wasilewska A. Vlad F. Valon C. Leung J. (2009). The guard cell as a single-cell model towards understanding drought tolerance and abscisic acid action. *J. Exp. Bot.* 60 1439–1463. 10.1093/jxb/ern340 19181866

[B48] Stone S. L. (2014). The role of ubiquitin and the 26S proteasome in plant abiotic stress signaling. *Front. Plant Sci.* 5:135 10.3389/fpls.2014.00135 PMC3997020 24795732

[B49] Stone S. L. Hauksdottir H. Troy A. Herschleb J. Kraft E. Callis J. (2005). Functional analysis of the RING-type ubiquitin ligase family of Arabidopsis. *Plant Physiol.* 137 13–30. 10.1104/pp.104.052423 15644464 PMC548835

[B50] Urano K. Maruyama K. Ogata Y. Morishita Y. Takeda M. Sakurai N. (2009). Characterization of the ABA-regulated global responses to dehydration in Arabidopsis by metabolomics. *Plant J.* 57 1065–1078. 10.1111/j.1365-313X.2008.03748.x 19036030

[B51] Verslues P. E. Bray E. A. (2006). Role of abscisic acid (ABA) and *Arabidopsis thaliana* ABA-insensitive loci in low water potential-induced ABA and proline accumulation. *J. Exp. Bot.* 57 201–212. 10.1093/jxb/erj026 16339784

[B52] Vierstra R. D. (2009). The ubiquitin-26S proteasome system at the nexus of plant biology. *Nat. Rev. Mol. Cell Biol.* 10 385–397. 10.1038/nrm2688 19424292

[B53] Vlad F. Rubio S. Rodrigues A. Sirichandra C. Belin C. Robert N. (2009). Protein phosphatases 2C regulate the activation of the Snf1-related kinase OST1 by abscisic acid in *Arabidopsis*. *Plant Cell* 21 3170–3184. 10.1105/tpc.109.069179 19855047 PMC2782292

[B54] Wu Q. Zhang X. Peirats-Llobet M. Belda-Palazon B. Wang X. Cui S. (2016). Ubiquitin ligases RGLG1 and RGLG5 regulate abscisic acid signaling by controlling the turnover of phosphatase PP2CA. *Plant Cell* 28 2178–2196. 10.1105/tpc.16.00364 PMC5059804 27577789

[B55] Yamaguchi-Shinozaki K. Shinozaki K. (2006). Transcriptional regulatory networks in cellular responses and tolerance to dehydration and cold stresses. *Annu. Rev. Plant Biol.* 57 781–803. 10.1146/annurev.arplant.57.032905.105444 16669782

[B56] Yang Y. Fu D. Zhu C. He Y. Zhang H. Liu T. (2015). The RING-finger ubiquitin ligase HAF1 mediates heading date 1 degradation during photoperiodic flowering in rice. *Plant Cell* 27 2455–2468. 10.1105/tpc.15.00320 26296966 PMC4815093

[B57] Zhang H. Cui F. Wu Y. Lou L. Liu L. Tian M. (2015). The RING finger ubiquitin E3 ligase SDIR1 targets SDIR1-INTERACTING PROTEIN1 for degradation to modulate the salt stress response and ABA signaling in *Arabidopsis*. *Plant Cell* 27 214–227. 10.1105/tpc.114.134163 25616872 PMC4330582

[B58] Zhang J. H. Jia W. S. Yang J. C. Ismail A. M. (2006). Role of ABA in integrating plant responses to drought and salt stresses. *Field Crops Res.* 97 111–119. 10.1016/j.fcr.2005.08.018

[B59] Zhu J. K. (2002). Salt and drought stress signal transduction in plants. *Annu. Rev. Plant Biol.* 53 247–273. 10.1146/annurev.arplant.53.091401.143329 12221975 PMC3128348

