# Supplementary material for: The Pepper RING Finger E3 Ligase, CaDIR1, Regulates the Drought Stress Response via ABA-Mediated Signaling
Source: Front Plant Sci. 2017 Apr 28;8:690. doi: 10.3389/fpls.2017.00690 (PMC5408085; doi:10.3389/fpls.2017.00690)
Supplement: Supplementary file 1 [file Image_1.PDF]

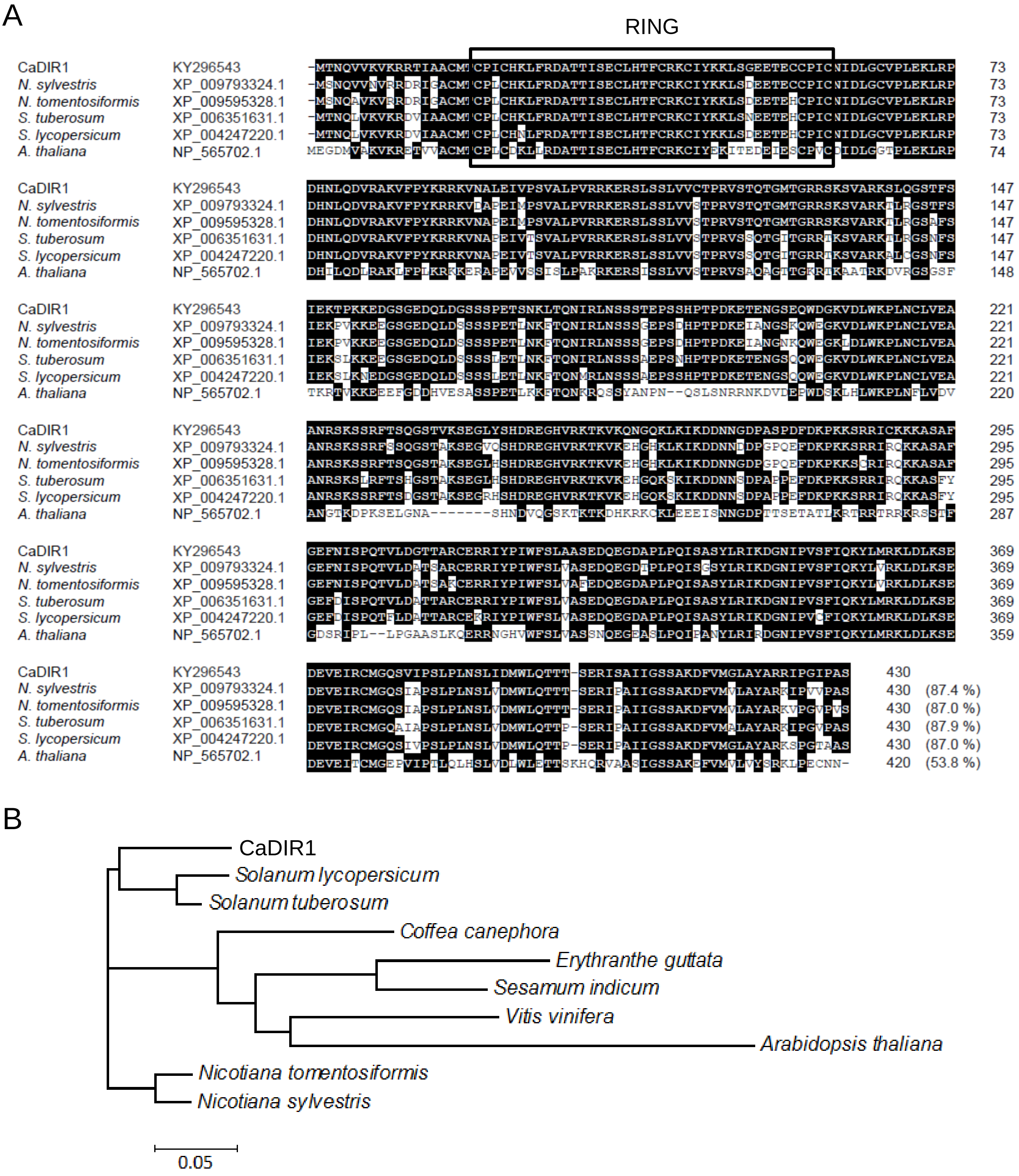

Supplementary Fig. S1. Joo et al.
