# Supplementary figures and images for: The Pepper RING Finger E3 Ligase, CaDIR1, Regulates the Drought Stress Response via ABA-Mediated Signaling
Source: Front Plant Sci. 2017 Apr 28;8:690. doi: 10.3389/fpls.2017.00690 (PMC5408085; doi:10.3389/fpls.2017.00690)

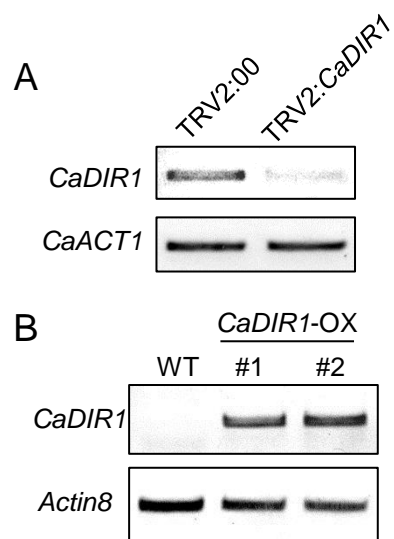

Supplement: Supplementary file 2 [file Image_2.PDF]
