# Supplementary material for: The Pepper RING Finger E3 Ligase, CaDIR1, Regulates the Drought Stress Response via ABA-Mediated Signaling
Source: Front Plant Sci. 2017 Apr 28;8:690. doi: 10.3389/fpls.2017.00690 (PMC5408085; doi:10.3389/fpls.2017.00690)
Supplement: Supplementary file 3 [file Table_1.PDF]

Supplementary Table 1. Sequences of primers used in this study

Primer name	Primer sequence (5'-3')
For cloning	
CaDIR1	Forward: ATGACGAATCAAGTAGTGAAGGTGAA<br>Reverse: TCAAGAAGCCGGAATGCCTG
For RT-PCR	
CaDIR1	Forward: TGACGAATCAAGTAGTGAAGGTGAAGA<br>Reverse: TGAAGGCACAATTTCAAGTGCATT
CaACT1	Forward: GACGTGACCTAACTGATAACCTGAT<br>Reverse: CTCTCAGCACCAATGGTAATAACTT
Actin8	Forward: CAACTATGTTCTCAGGTATTGCAGA<br>Reverse: GTCATGGAAACGATGTCTCTTTAGT
RD20	Forward: TGGTTTCCTATCTAAAGAAGCTGTG<br>Reverse: ATACAAATCCCCAAACTGAATAACA
RD26	Forward: AGGTCTTAATCCAATTCCAGAGCTA<br>Reverse: ACCCATCAGTAACTTCACATCTCTC
RD29B	Forward: GTTGAAGAGTCTCCACAATCACTTG<br>Reverse: ATACAAATCCCCAAACTGAATAACA
DREB2A	Forward: CTACAAAGCCTCAACTACGGAATAC<br>Reverse: AAACTCGGATAGAGAATCAACAGTC
NCED3	Forward: ACATGGAAATCGGAGTTACAGATAG<br>Reverse: AGAAACAACAAACAAGAAACAGAGC
RAB18	Forward: GGAAGAAGGGAATAACACAAAAGAT<br>Reverse: GCGTTACAAACCCTCATTATTTTTA
COR47	Forward : ACAGAGGTTACGGATCGTGGA<br>Reverse : ACTCGAGCGTCGTTGTCTCTT
ERD10	Forward : TCATCGGCGCCAGAGATTAA<br>Reverse : GCCACAAACGACTCTGGTTCA
LTI30	Forward : TCAAACCGGAGTGCAAAAGAAG<br>Reverse : CGGTACCAGTAGCACCATGATG
